# Supplementary material for: HSD17B7 is required for the function of sensory hair cells by regulating cholesterol synthesis
Source: eLife. 2026 Jun 3;14:RP108108. doi: 10.7554/eLife.108108 (PMC13233068; doi:10.7554/eLife.108108)
Supplement: Figure 1—figure supplement 1—source data 2. [file elife-108108-fig1-figsupp1-data2.pdf]

I

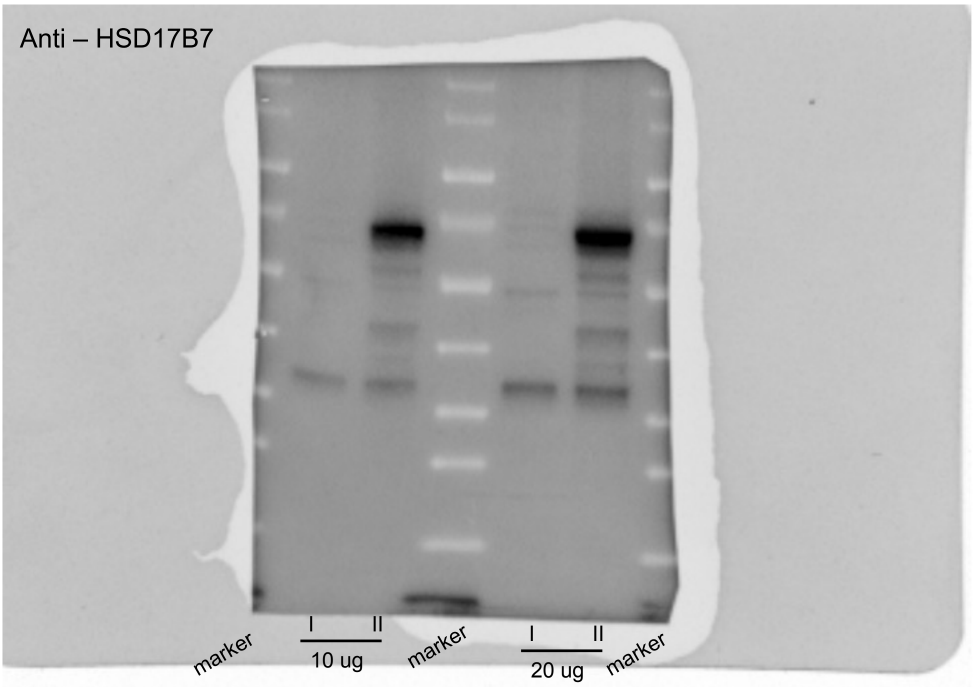

II

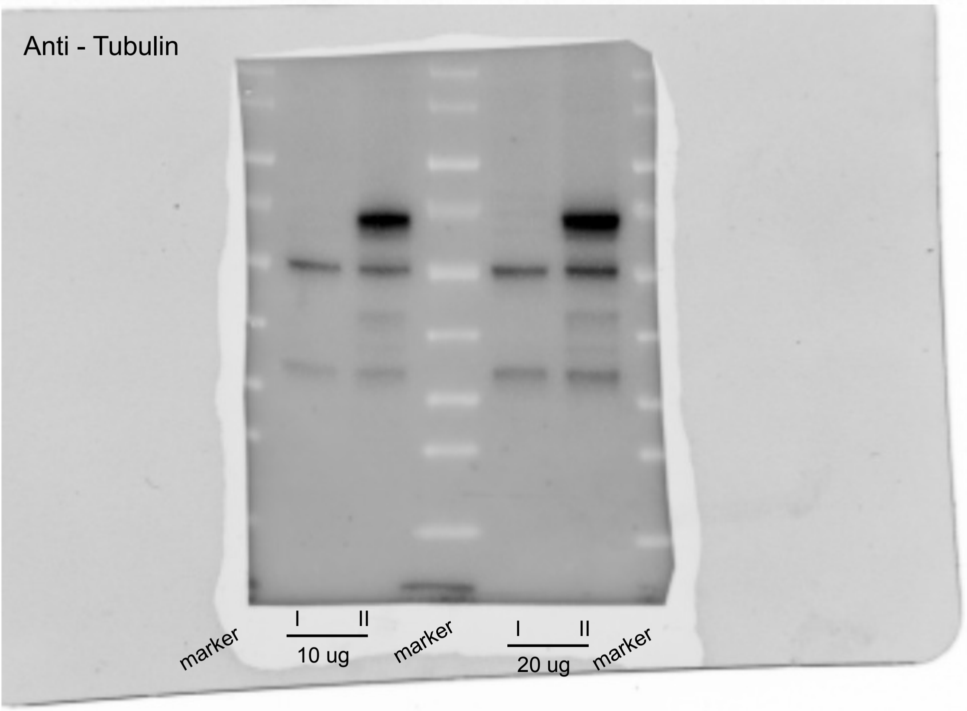

Figure 1-figure supplement1-source data 1. Original membranes corresponding to Figure 1-figure supplement1B. Rainbow molecular weight markers were employed. After overexpressing GFP and the HSD17B7-GFP plasmid, the membrane was first incubated with an HSD17B7 antibody for detection, followed by incubation and detection with a tubulin antibody. Panel I shows the detection results using the HSD17B7 antibody, and marker shows the corresponding protein marker results. Panel II shows the detection results using the tubulin antibody, and marker shows the corresponding protein marker results.
