# Supplementary figures and images for: HSD17B7 is required for the function of sensory hair cells by regulating cholesterol synthesis
Source: eLife. 2026 Jun 3;14:RP108108. doi: 10.7554/eLife.108108 (PMC13233068; doi:10.7554/eLife.108108)

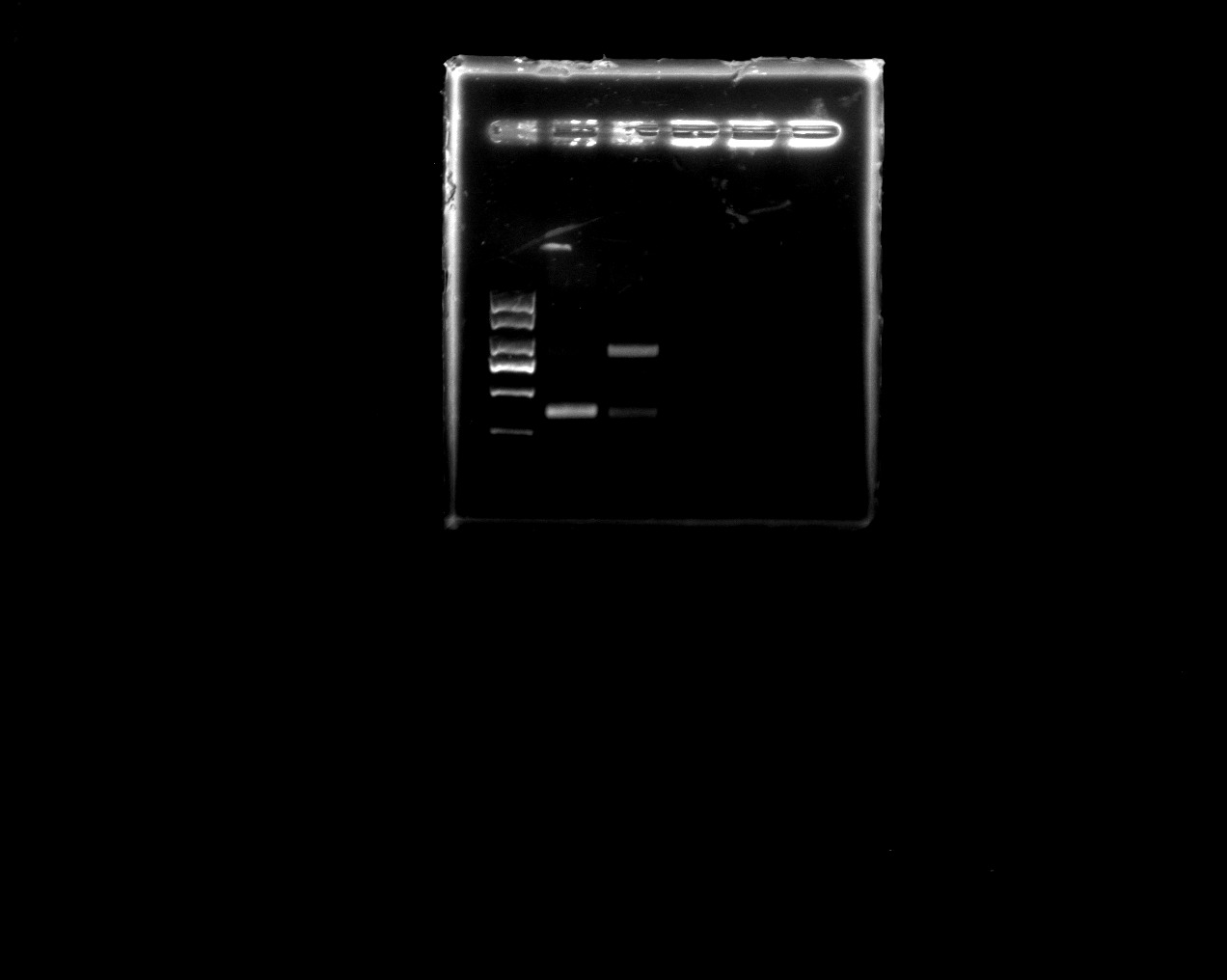

Supplement: Figure 2—figure supplement 2—source data 1. [file elife-108108-fig2-figsupp2-data1.zip › figure 2-figure supplement 2-source data 1/hsd17b7-Mo-PCR.jpg]

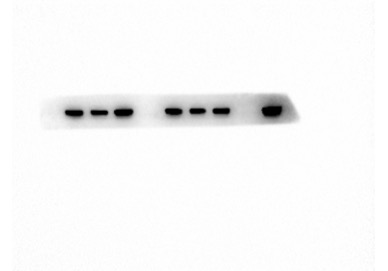

Supplement: Figure 2—figure supplement 2—source data 3. [file elife-108108-fig2-figsupp2-data3.zip › Figure 2-figure supplement 2-source data 2/Anti-Tubulin.tif]

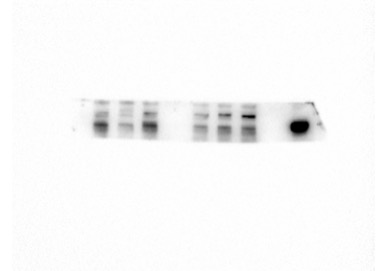

Supplement: Figure 2—figure supplement 2—source data 3. [file elife-108108-fig2-figsupp2-data3.zip › Figure 2-figure supplement 2-source data 2/Anti-Hsd17b7.tif]

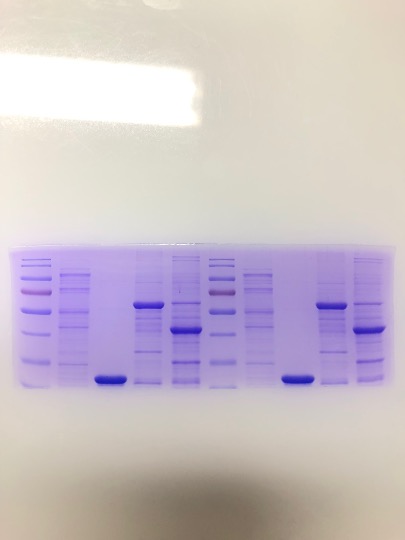

Supplement: Figure 9—source data 3. [file elife-108108-fig9-data3.zip › Figure 9-source data 2/Coomassie blue staining.jpg]

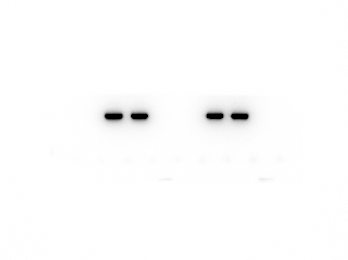

Supplement: Figure 9—figure supplement 2—source data 1. [file elife-108108-fig9-figsupp2-data1.zip › figure 9-figure supplement 2-source data 1/Anti-Flag.scn]

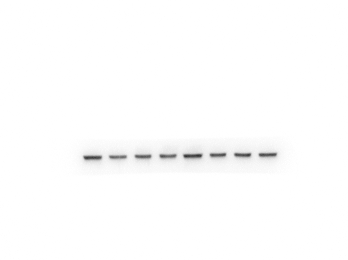

Supplement: Figure 9—figure supplement 2—source data 1. [file elife-108108-fig9-figsupp2-data1.zip › figure 9-figure supplement 2-source data 1/Anti-Tubulin.scn]
