# Supplementary material for: HSD17B7 is required for the function of sensory hair cells by regulating cholesterol synthesis
Source: eLife. 2026 Jun 3;14:RP108108. doi: 10.7554/eLife.108108 (PMC13233068; doi:10.7554/eLife.108108)
Supplement: Figure 2—figure supplement 2—source data 2. [file elife-108108-fig2-figsupp2-data2.pdf]

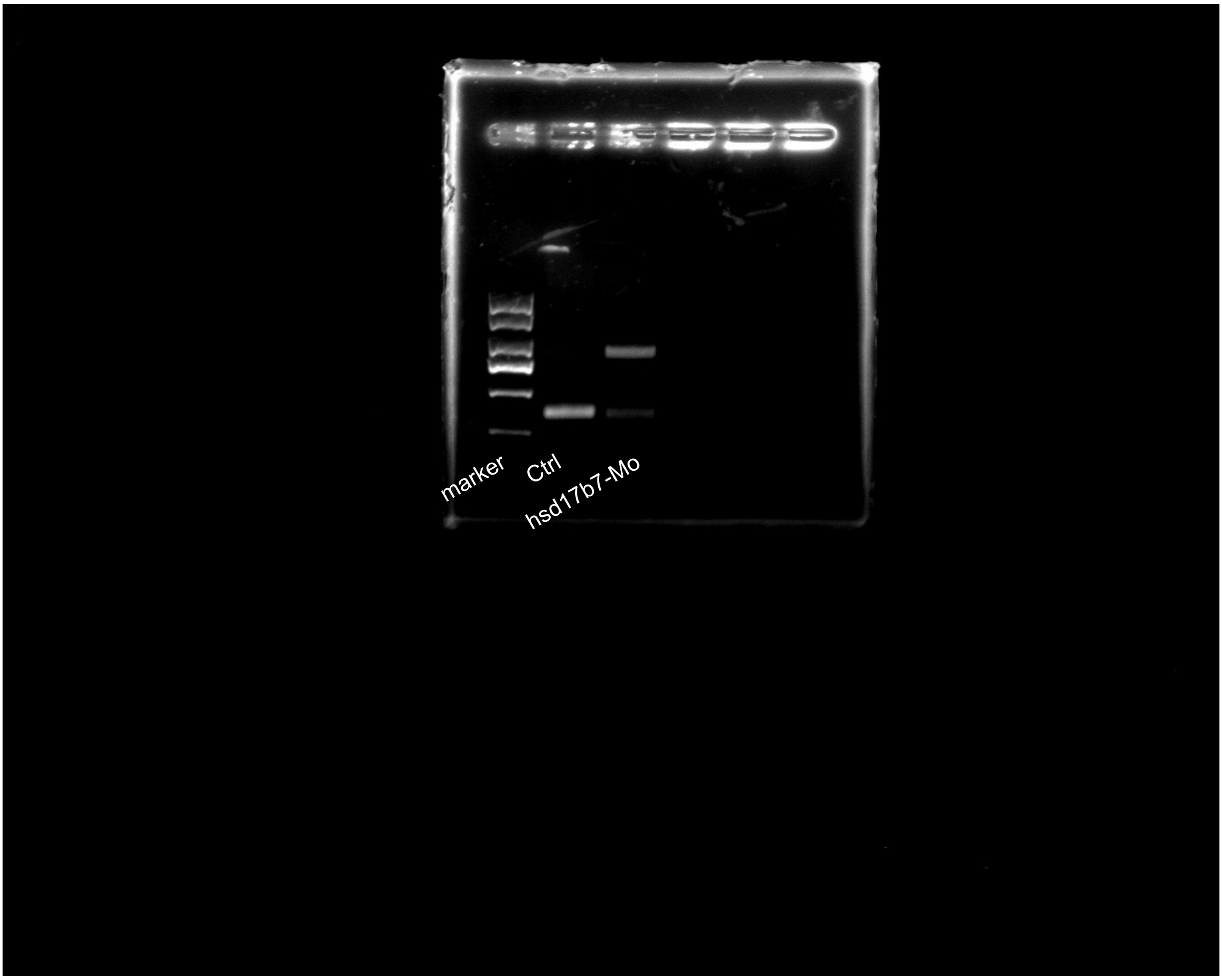


**Figure 2–figure supplement 2-source data 1.** Original membranes corresponding to Figure 2–figure supplement 2C. DNA markers were employed.
