# Supplementary material for: HSD17B7 is required for the function of sensory hair cells by regulating cholesterol synthesis
Source: eLife. 2026 Jun 3;14:RP108108. doi: 10.7554/eLife.108108 (PMC13233068; doi:10.7554/eLife.108108)
Supplement: Figure 2—figure supplement 2—source data 4. [file elife-108108-fig2-figsupp2-data4.pdf]

I

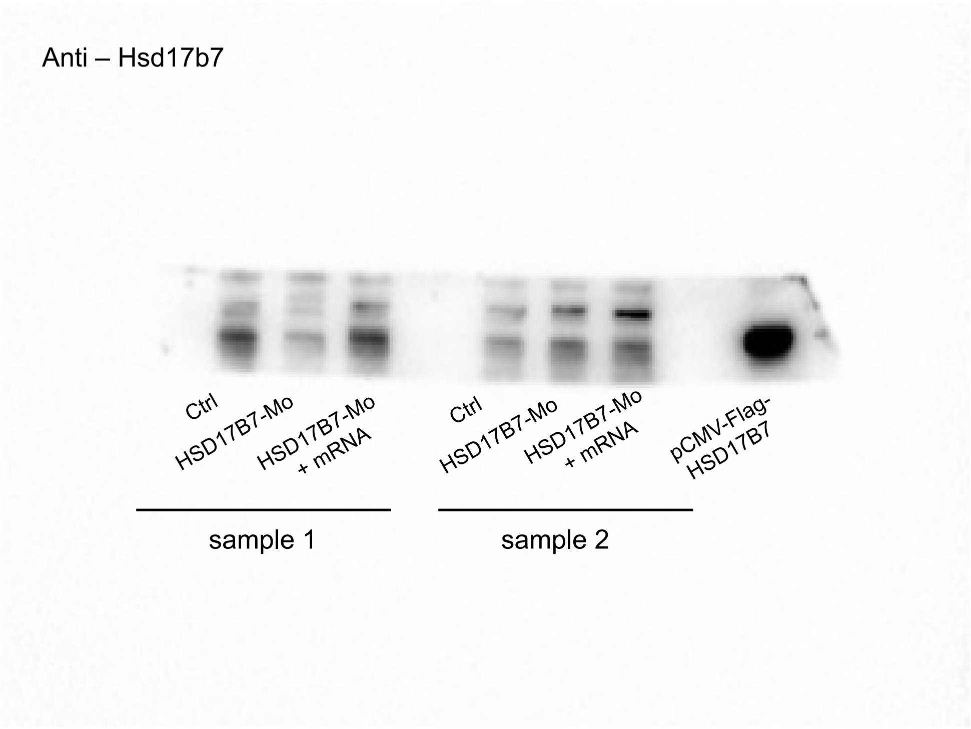

II

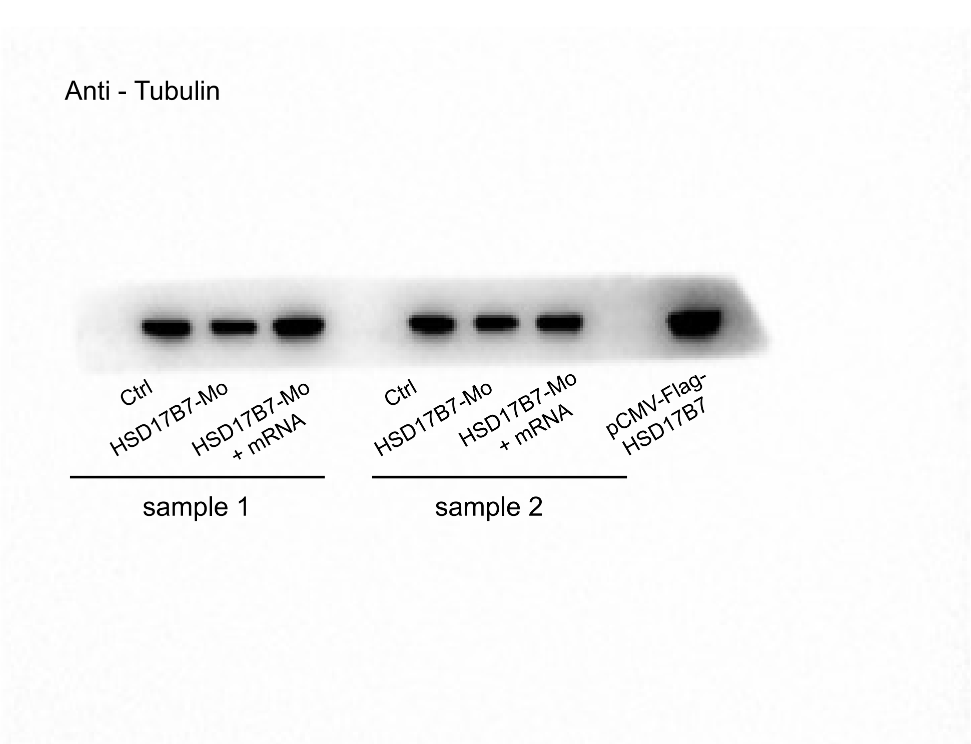

Figure 2–figure supplement 2-source data 2. Original membranes corresponding to **Figure 2–figure supplement 2D**. Lanes 1 and 5 represent the negative control group, lanes 2 and 6 represent the HSD17B7 MO-treated group, lanes 3 and 7 represent the HSD17B7 MO + mRNA treated group, lanes 4 and 8 were empty. Lane 9 was injected with the pCMV-Flag-HSD17B7 plasmid as a positive control. Rainbow molecular weight markers were employed. Panel **I** shows the detection results using the HSD17B7 antibody. Panel **II** shows the detection results using the tubulin antibody. Lanes 1, 2, and 3 are shown in Figure S4D of the article.
