# Supplementary material for: HSD17B7 is required for the function of sensory hair cells by regulating cholesterol synthesis
Source: eLife. 2026 Jun 3;14:RP108108. doi: 10.7554/eLife.108108 (PMC13233068; doi:10.7554/eLife.108108)
Supplement: Figure 4—source data 2. [file elife-108108-fig4-data2.pdf]

I

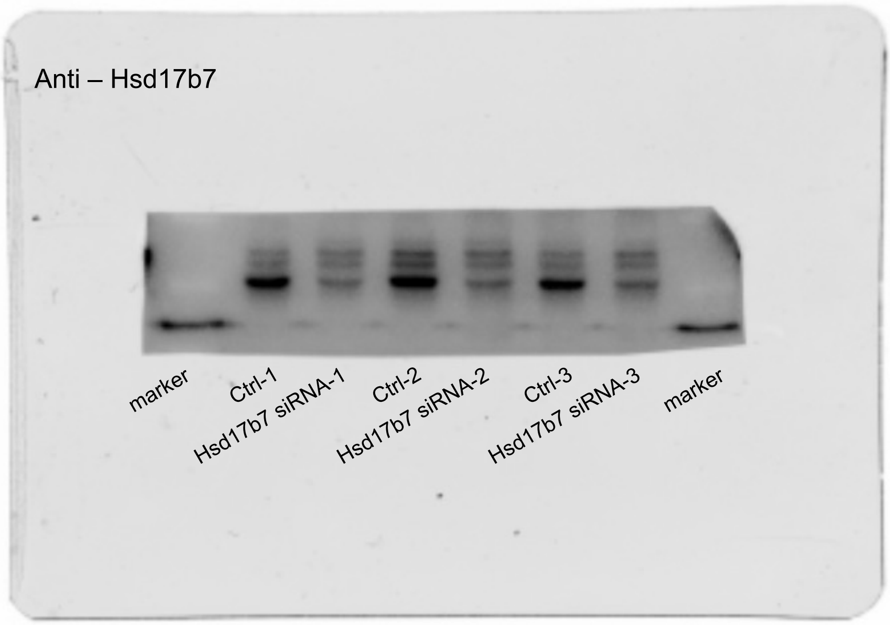

II

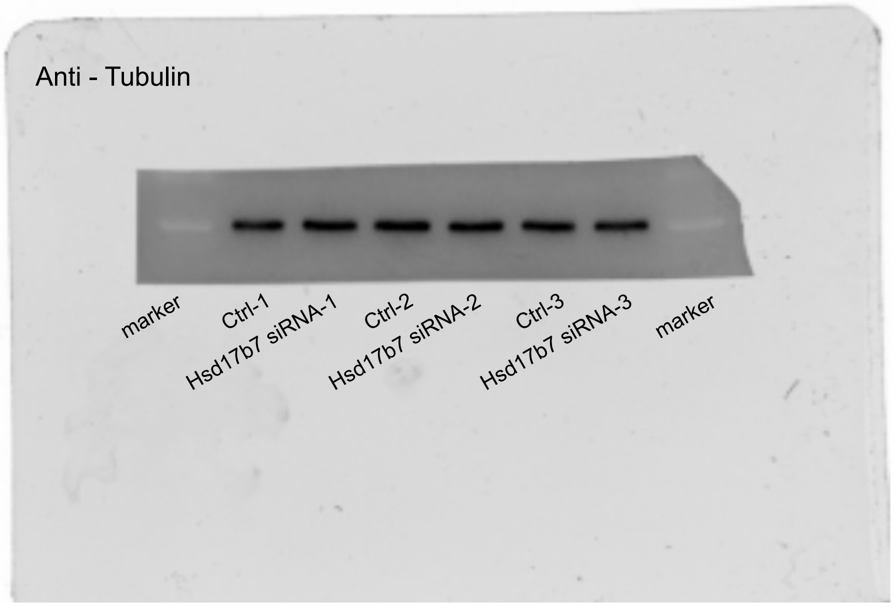

Figure 4 - sourse data 1. Original membranes corresponding to Figure 4B. Lanes 1, 3, and 5 represent the negative control group, while lanes 2, 4, and 6 represent the HSD17B7 siRNA-treated group. Rainbow molecular weight markers were employed. Panel I shows the knockdown efficiency detected using an HSD17B7 antibody, and marker shows the corresponding protein marker results. Panel II shows the detection of the loading control tubulin, and marker shows the corresponding protein marker image. Lanes 3 and 4 are shown in Figure 4B of the article.
