# Supplementary material for: HSD17B7 is required for the function of sensory hair cells by regulating cholesterol synthesis
Source: eLife. 2026 Jun 3;14:RP108108. doi: 10.7554/eLife.108108 (PMC13233068; doi:10.7554/eLife.108108)
Supplement: Figure 9—source data 2. [file elife-108108-fig9-data2.pdf]

I

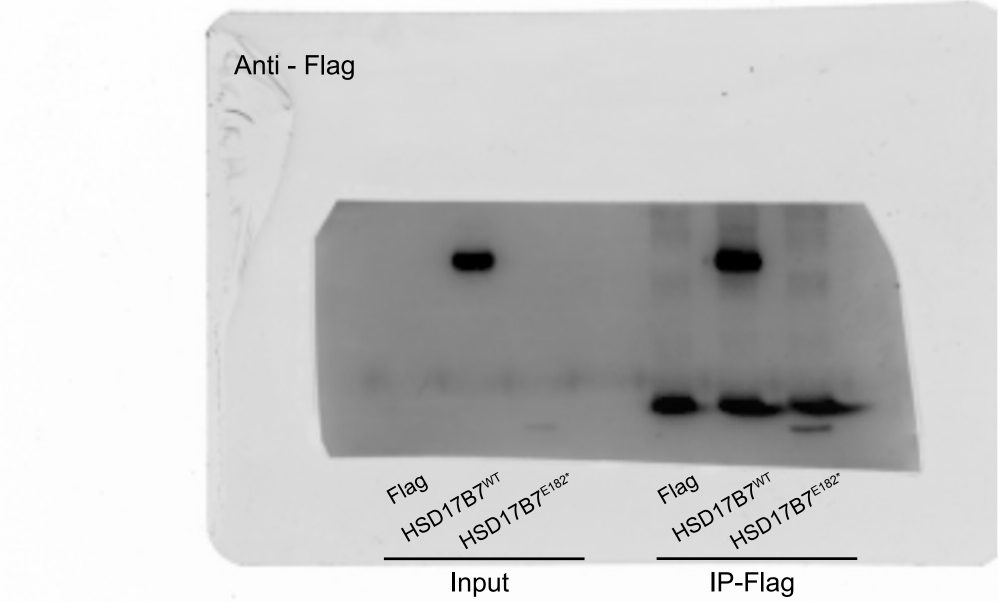

II

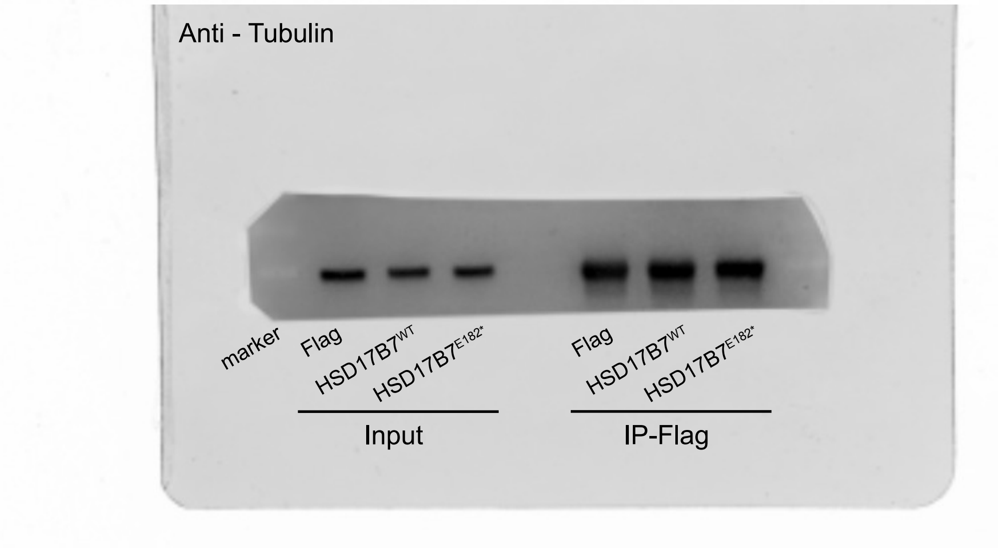

Figure 9-source data 1. Original membranes corresponding to Figure 9A. Rainbow molecular weight markers were employed. Panel I shows the detection results using the FLAG antibody, and marker shows the corresponding protein marker results. Panel II shows the detection results using the tubulin antibody, and marker shows the corresponding protein marker results.
