# Supplementary material for: HSD17B7 is required for the function of sensory hair cells by regulating cholesterol synthesis
Source: eLife. 2026 Jun 3;14:RP108108. doi: 10.7554/eLife.108108 (PMC13233068; doi:10.7554/eLife.108108)
Supplement: Figure 9—source data 4. [file elife-108108-fig9-data4.pdf]

I

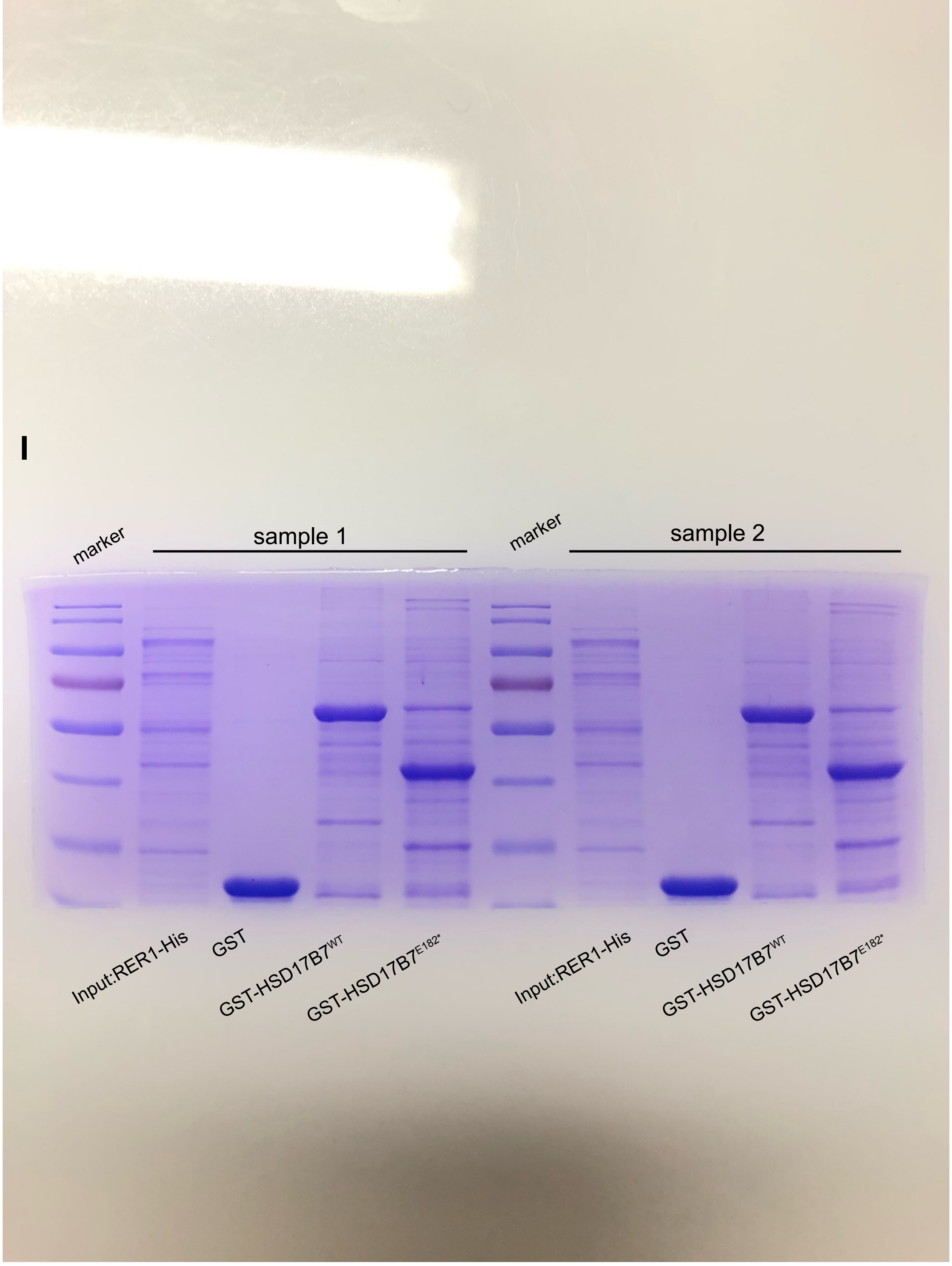

II

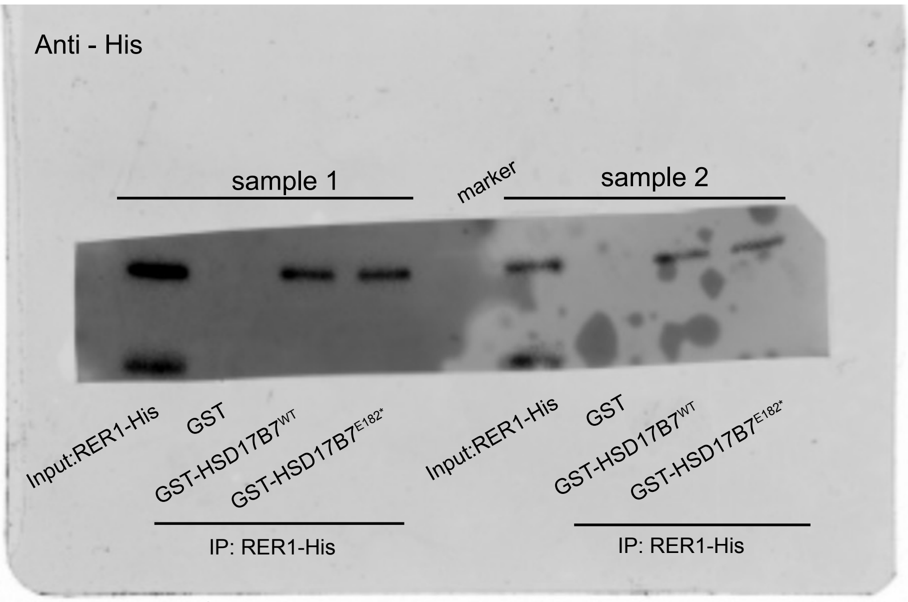

Figure 9-source data 2. Original membranes corresponding to Figure 9F. Rainbow molecular weight markers were employed. Panel I shows Coomassie blue staining of purified GST and GST-HSD17B7 proteins. Panel II shows the detection results using the His antibody, and I-marker shows the corresponding protein marker results.
