# Supplementary material for: HSD17B7 is required for the function of sensory hair cells by regulating cholesterol synthesis
Source: eLife. 2026 Jun 3;14:RP108108. doi: 10.7554/eLife.108108 (PMC13233068; doi:10.7554/eLife.108108)
Supplement: Figure 9—figure supplement 2—source data 2. [file elife-108108-fig9-figsupp2-data2.pdf]

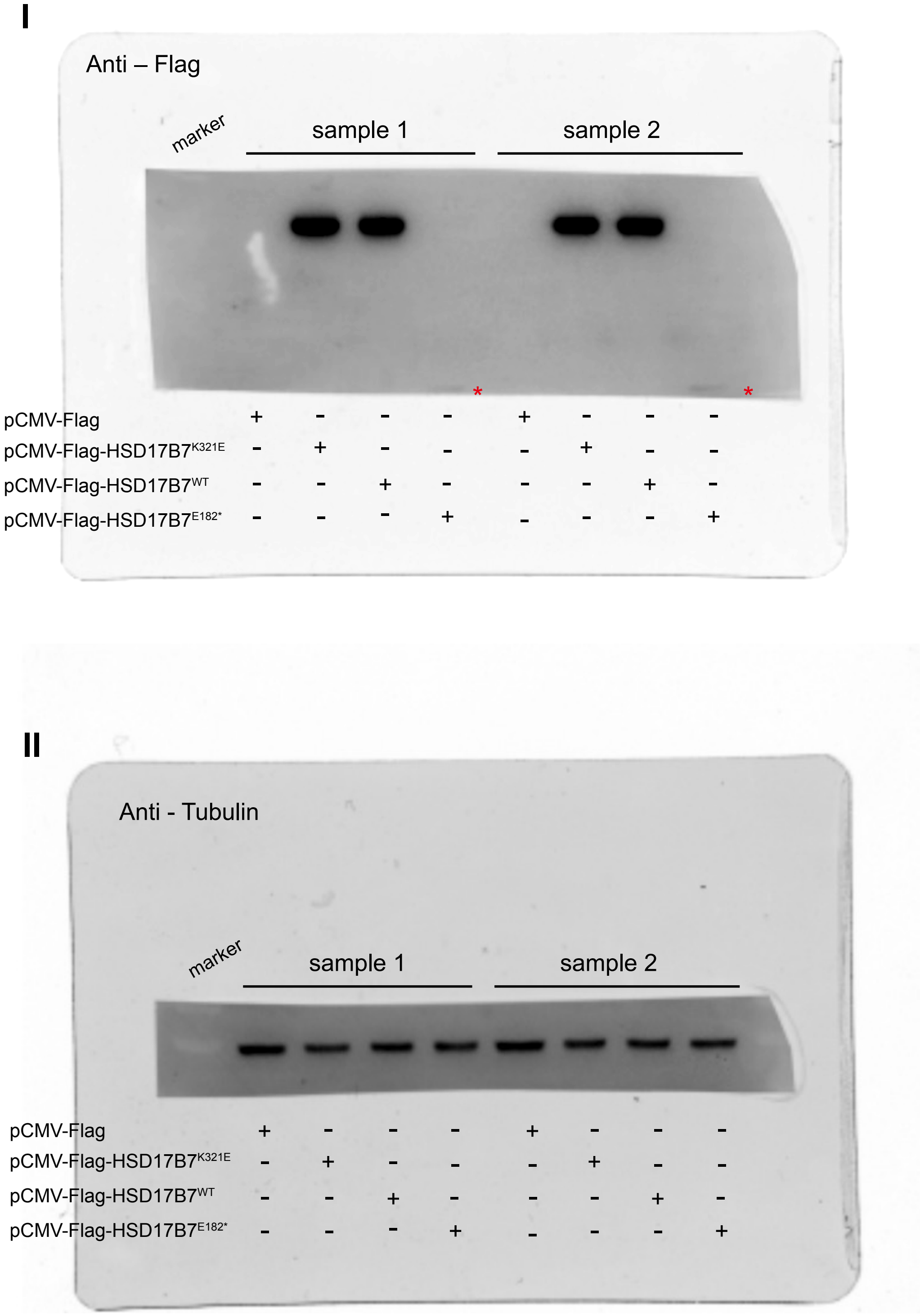

Figure 9–figure supplement 2-source data 1. Original membranes corresponding to Figure 9–figure supplement 2B. Lanes 1, 2, 3, and 4 correspond to the first experiment, in which cells were transfected with FLAG, FLAG-HSD17B7K32E, FLAG-HSD17B7WT, and FLAG-HSD17B7E182* plasmids, respectively. Lanes 5, 6, 7, and 8 correspond to the second experiment, in which cells were transfected with FLAG, FLAG-HSD17B7K32E, FLAG-HSD17B7WT, and FLAG-HSD17B7E182* plasmids, respectively. Rainbow molecular weight markers were employed. Panel I shows the detection results using the flag antibody, and marker shows the corresponding protein marker results. Panel II shows the detection results using the tubulin antibody, and marker shows the corresponding protein marker results. Lanes 3 and 4 are shown in Figure S8B of the article.
